# An Integrated Microfluidic Biosensing System Based on a Versatile Valve and Recombinase Polymerase Amplification for Rapid and Sensitive Detection of *Salmonella typhimurium*

**DOI:** 10.3390/bios13080790

**Published:** 2023-08-04

**Authors:** Yan Jin, Jingyi Wang, Zhiqiang Wang, Peng Xiong, Jianing Cheng, Tongyu Xu

**Affiliations:** 1 College of Information and Electrical Engineering, Shenyang Agricultural University, Shenyang 110866, China; jinyan@stu.syau.edu.cn (Y.J.);; 2 Liaoning Engineering Research Center for Information Technology in Agriculture, Shenyang 110866, China; 3 College of Bioscience and Biotechnology, Shenyang Agricultural University, Shenyang 110866, China

**Keywords:** microfluidic chip, RPA, versatile valve, *Salmonella* detection

## Abstract

Detecting foodborne pathogens on-site is crucial for ensuring food safety, necessitating the development of rapid, cost-effective, highly sensitive, and portable devices. This paper presents an integrated microfluidic biosensing system designed for the rapid and sensitive detection of *Salmonella typhimurium* (*S. typhimurium*). The biosensing system comprises a microfluidic chip with a versatile valve, a recombinase polymerase amplification (RPA) for nucleic acid detection, and a customized real-time fluorescence detection system. The versatile valve combines the functions of an active valve and a magnetic actuation mixer, enabling on-demand mixing and controlling fluid flow. Quantitative fluorescence is processed and detected through a custom-built smartphone application. The proposed integrated microfluidic biosensing system could detect *Salmonella* at concentrations as low as 1.0 × 10^2^ copies/µL within 30 min, which was consistent with the results obtained from the real-time quantitative polymerase chain reaction (qPCR) tests. With its versatile valve, this integrated microfluidic biosensing system holds significant potential for on-site detection of foodborne pathogens.

## 1. Introduction

Food safety is a significant challenge to human health and is a primary concern in food production. Foodborne pathogens precipitate a destructive cycle of disease and malnutrition, particularly impacting infants, the elderly, and patients with compromised immunity. Statistics from the Centers for Disease Control and Prevention reveal that annually, 48 million individuals fall ill and 3000 die due to foodborne illnesses [1]. Effective and rapid detection of foodborne pathogens is therefore pivotal in mitigating the spread of foodborne illnesses [2]. Current methods for foodborne pathogen detection include traditional plate colony counting, polymerase chain reaction (PCR), and enzyme-linked immunosorbent assay (ELISA). However, the plate colony counting method (the gold standard) is labor-intensive and time-consuming, often requiring 24–48 h [3,4]. PCR can offer accurate detection through the exponential amplification of the target DNA, and ELISA provides visual detection based on antigen–antibody recognition and enzyme biocatalytic properties [5]. However, both PCR and ELISA require expensive equipment and may yield false-positive results [6,7,8,9]. Consequently, the urgent need for reliable, sensitive, and rapid detection methods as alternatives to traditional time-consuming procedures is apparent [10]. Therefore, isothermal amplification techniques have been proposed for the detection of foodborne pathogens. By eliminating complex thermal cycling processes, these techniques can simplify the operation and reduce detection time. Isothermal amplification methods such as loop-mediated isothermal amplification (LAMP) [11,12], rolling circle amplification (RCA) [13,14], strand displacement amplification (SDA) [15,16], and recombinase polymerase amplification (RPA) [17,18] offer the benefits of low cost, high sensitivity, and high specificity. Of these, RPA has demonstrated significant potential for practical applications due to its capacity to perform rapid detection in under 40 min at a consistent temperature of 37–40 °C, by introducing recombinase, strand-substitution polymerase, and ssDNA-binding protein (SSB) [19,20]. Rapid and sensitive detection by using RPA has become a subject of intense recent focus. Chen et al., for instance, used fluorescent probes to monitor the entire RPA reaction process of *Salmonella* in real-time, with a detection limit of 1.0 × 10^2^ copies/reaction [21]. In another study, Guo et al. successfully detected *Listeria monocytogenes* (*L. monocytogenes*) and *Staphylococcus aureus* (*S. aureus*) simultaneously at concentrations as low as 1.0 × 10^2^ copies/µL in under 15 min RPA analysis [22].

Microfluidic chip systems [23,24,25] have a substantial amount of attention due to their compact size, reduced reagent consumption, automatic operation, and rapid detection capabilities, establishing them as an ideal on-site platform for the swift detection of foodborne pathogens [26,27,28]. Currently, the RPA analysis based on various microfluidic chips is extensively employed for the rapid and precise detection of such pathogens [29,30,31]. For instance, Xia et al. used a combination of RPA-CRISPR/Cas12a and a microfluidic chip to detect *Salmonella* at concentrations as low as 0.2 cells/mL within 30 min [32]. Yin et al. proposed an integrated multiplex digital RPA microfluidic chip that included DNA extraction, multiplex RPA reaction, and fluorescence detection, capable of simultaneously detecting *Escherichia coli* O157:H7 (*E. coli*), *S. typhimurium*, and *L. monocytogenes* within 15 min [33]. However, the loss and flow of liquid may occur during the heating process of the isothermal amplification method. Addressing this issue effectively is critical for enhancing detection accuracy. Rapid and efficient mixing plays an instrumental role in various microfluidic chip systems [34]. Moreover, the tightness of the mixing chamber affects the reaction efficiency. Therefore, efforts are currently underway, whether through active or passive mixing, to develop innovative micromixers [35,36,37,38].

**Figure 1:**
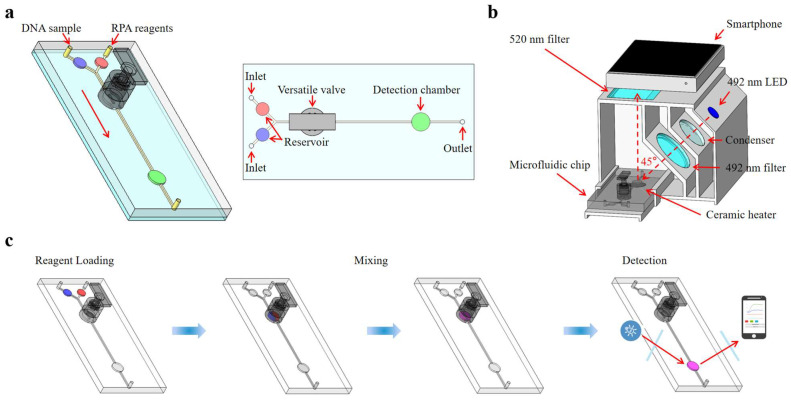
Schematic diagram of the microfluidic biosensing system for *Salmonella* detection. (**a**) The schematic diagram of the microfluidic chip. (**b**) The structure of the real-time fluorescence detection system. (**c**) The whole procedure for *Salmonella* detection.

This paper presents an integrated microfluidic biosensing system designed for the quantitative detection of foodborne pathogens based on versatile valves for liquid mixing, RPA for nucleic acid detection, a temperature control module for heating amplification, and a real-time fluorescence detection system for signal analysis. A novel versatile valve is proposed, which combines an active valve and a magnetic actuation mixer, facilitating on-demand mixing and fluid flow control. We devised a fluorescence detection system for the quantitative fluorescence detection of RPA reactions, incorporating a 3D-printed detection box with optical filters, an LED source, and a ceramic heater. Quantitative fluorescence results were read and analyzed using a custom-built smartphone app. As shown in Figure 1, the *Salmonella* DNA sample and RPA reagents were automatically loaded into the microfluidic chip using a syringe pump and fully mixed in the versatile valve. After being transferred into the detection chamber, the mixture was isothermally amplified at 40 °C and real-time monitored by the fluorescence detection system. The sample loading, mixing, and detection were performed automatically by using a microcontroller (STM32F103). The photo of the integrated microfluidic biosensing system is shown in Figure S1. Through all these innovations, the proposed microfluidic biosensing system could detect *S. typhimurium* at concentrations as low as 1.0 × 10^2^ copies/µL within 30 min, aligning with the real-time RPA results from qPCR instruments. Consequently, this microfluidic biosensing system could serve as a rapid detection tool for *Salmonella* and other foodborne pathogens.

## 2. Materials and Methods

### 2.1. Materials

*Salmonella typhimurium* (ATCC 14028) was used as the target bacteria, with *E. coli* O157:H7 (ATCC 43888), *Vibrio parahemolyticus* (ATCC 17802, *V. parahemolyticus*), and *Bacillus cereus* (ATCC 11778, *B. cereus*) served as non-target bacteria. Purified DNA from these foodborne pathogens was procured from Testo Biotech (Ningbo, China). The DNA was extracted from 5 × 10^7^ CFU/mL *S. typhimurium* by using SteadyPure Bacteria Genomic DNA Extraction Kit (Accuate Biotechnology Co., Ltd., Changsha, China). A commercial RPA detection kit for *S. typhimurium* was obtained from GenDx Biotech (GenDx Biotech Co., Ltd., Suzhou, China). A photosensitive resin provided by Formlabs (Formlabs Inc., Boston, MA, USA) was employed to fabricate microfluidic chip molds and versatile valves. Dow Corning (Dow Corning, Midland, MI, USA) supplied the polydimethylsiloxane (PDMS) used for producing the microfluidic chips.

### 2.2. Real-Time RPA Assay

The RPA assay was performed using a commercial RPA kit, according to the manufacturer’s instructions. Briefly, a 50 µL RPA system comprised 2 µL DNA sample, 2 µL magnesium acetate, 20 µL RPA basic buffer, 26 µL ddH_2_O, and a freeze-dried reagent powder (primarily including primers, FAM probes, etc.). After thorough mixing, the mixture was incubated in a qPCR instrument (LightCycler 480, Roche, Basel, Switzerland), with fluorescence signals collected every 30 s.

### 2.3. RPA Optimization

To optimize the RPA reaction temperature for *S. typhimurium*, 1.0 × 10^5^ copies/µL of the purified DNA was used as a template and was tested under various temperatures (38 °C, 39 °C, 40 °C, 41 °C, and 42 °C). To optimize the RPA detection time, 1.0 × 10^5^ copies/µL of the purified DNA was again used as a template, with the RPA reaction times performed across a range of 15, 20, and 25 min. The optimal reaction temperature and detection time for the RPA were determined based on the *C_p_* (crossing point) value.

### 2.4. Sensitivity and Specificity of RPA

Operating under optimal conditions, 10-fold serial dilutions of *S. typhimurium* purified DNA, ranging from 1.0 × 10^0^ copies/µL to 1.0 × 10^5^ copies/µL, were selected for limit of detection (LoD) analysis. *S. typhimurium* was identified as the target bacteria, while *E. coli*, *V. parahemolyticus*, and *B. cereus* functioned as non-target bacteria. Purified DNA from the four pathogens, each at the same concentration (1.0 × 10^5^ copies/µL), was employed for specificity analysis.

### 2.5. Design and Fabrication of the Microfluidic Chip

**Figure 2:**
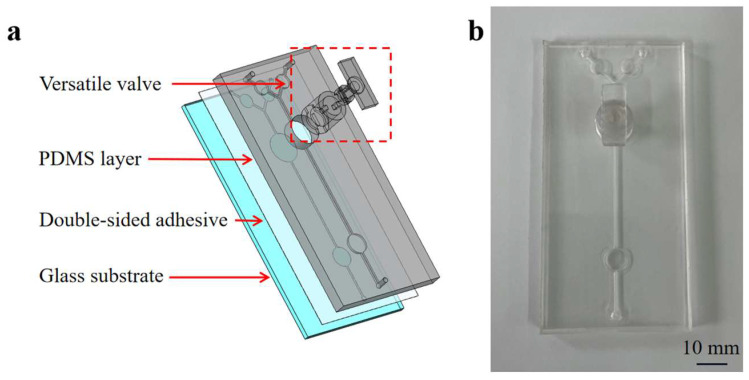
An exposed view of the chip (**a**) and an image of the microfluidic chip (**b**).

A microfluidic chip incorporating a versatile valve was developed to integrate mixing, RPA reactions, and detection. Figure 2 illustrates the components of the microfluidic chip, which include: (1) a glass substrate; (2) the PDMS layer (100 mm long × 50 mm wide × 5 mm high) containing two inlets, one outlet, two reservoirs (25 µL volume), one detection chamber (50 µL volume), and one channel (0.8 mm wide × 1 mm high) structure; (3) 3D-printed versatile valve components (designed with a convex and concave aligned structure and two magnetic bars) were used for on-demand mixing and controlling the flow of the fluid. The circular mixing chamber at the bottom of the valve can hold up to 50 µL of reagents.

The 3D-printed mold for the microfluidic chip and versatile valve was initially printed using a Form 3 printer from Formlabs (Formlabs Inc., Boston, MA, USA). Both the mold and versatile valve were cleaned with an isopropyl alcohol solution for 3 min and then soaked in the same solution for 30 min to thoroughly remove any residual resin material. Next, a PDMS prepolymer and curing agent (in a 10:1 mass ratio) were stirred together using a glass rod. This mixture was poured into the 3D mold and cured at 65 °C overnight following vacuum processing. The microfluidic chip was ultimately obtained as follows: detaching the PDMS from the 3D printed mold; bonding the PDMS to a glass substrate using a 140 μm thick laser-engraved double-sided pressure-sensitive adhesive (PSA) sheet (ARcare 90106NB, Adhesive Research Inc., Glen Rock, NJ, USA). According to the structure of the microfluidic chip, the PSA sheet was laser-cut by a laser engraving machine (Snapmaker Original, Snapmaker Co., Ltd., Shenzhen, China). After bonding the PDMS layer to a glass substrate, the mobile magnetic bar was placed into the reserved position of the mixing chamber in the PDMS layer. Then, the versatile valve was inserted into the reserved position in the PDMS layer. Finally, 15 μL mineral oil was used to cover the contact line between the versatile valve and the top surface of the PDMS layer to maintain the seal. Prior to use, the microfluidic chip was thoroughly washed with deionized water.

### 2.6. Versatile Valve System

**Figure 3:**
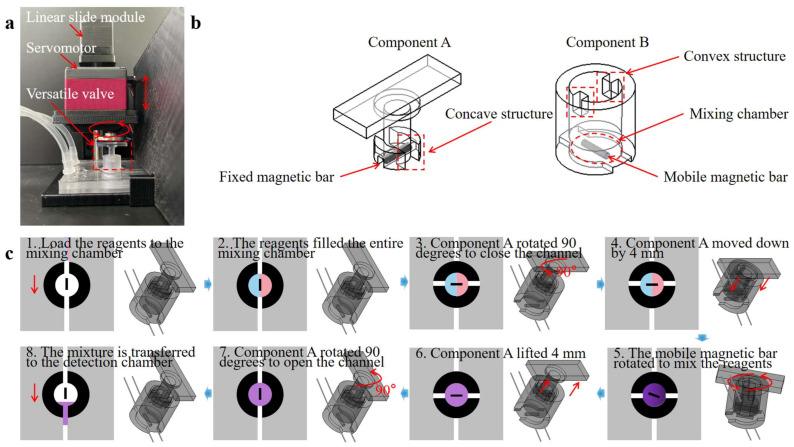
(**a**) An image of the versatile valve system. (**b**) Design scheme of the versatile valve design scheme. (**c**) Schematic illustration of working process of the versatile valve (1. load the reagents to the mixing chamber from inlets; 2. the reagents filled the entire mixing chamber; 3. Component A rotated 90 degrees to close the channel as a valve; 4. Component A moved down by 4 mm to disengage from the concave–convex alignment structure; 5. Component A rotated at a speed of 360 degrees per second for the mobile magnetic bar to stir the reagents; 6. after mixing, Component A lifted 4 mm to mesh the convex of Component B; 7. Component A rotated 90 degrees to open the channel; 8. the mixture of the reagents is transferred to the detection chamber for RPA detection).

As shown in Figure 3a, the versatile valve comprises two parts: a rotational mixing part (Component A) and a valve part (Component B). Figure 3b showcases a detailed valve design scheme. Component A is used for magnetic actuation mixing and valve control, with a magnetic bar fixed at its base. It is designed with a concave structure to mesh with the convex Component B, thereby driving valve rotation and regulating the flow. Component B also serves as a mixing chamber, with a mobile magnetic mixing bar placed within. The versatile valve system incorporates a microcontroller unit (MCU) controller, drive module, power module, linear slide module, and a servomotor. Figure 3c and Video S1 demonstrate the versatile valve’s workflow as follows: Component A and Component B are connected through the concave–convex alignment structure in the initial state. (1) load the reagents into the mixing chamber through the opening of Component B; (2) after the reagents fill the entire mixing chamber; (3) Component A drives Component B to rotate 90° to seal the chamber as a valve; (4) Component A descends by 4 mm to disengage from the concave–convex alignment structure and rotates at a speed of 360° per second; (5) the mobile magnetic bar rotates to mix the reagents owing to the magnetic torque of the fixed magnetic bar in Component A.

(1) T=VM×B

where V is the volume of the mobile magnetic bar; M is the magnetization vector of the bar; and B is the magnetic flux vector generated by the fixed magnetic bar of Component A; (6) after mixing, Component A is lifted by 4 mm to engage with the convex of Component B; (7) and drives Component B to rotate 90° to open the channel; (8) the reagents mixture is then transferred through the opening of Component B to the detection chamber for RPA detection. Before loading the reagents, Component A is controlled to rotate the mobile magnetic bar in the direction of the channel. Detailed information on components A and B is provided in the Supplementary File (Figure S2).

### 2.7. Temperature Control System and Real-Time Fluorescence Detection System

The RPA temperature control system incorporates an MCU controller, a drive module, a circular ceramic heater, and a temperature sensor. To ensure rapid and uniform heating of the detection chamber, we selected a ceramic heater and a Pt100 temperature sensor to maintain a constant temperature and acquire data. The ceramic heater was controlled by the microcontroller and powered by a 5 V external power supply (D-30A, Junlin Electric Co., Ltd., Wenzhou, China). Using the PID (Proportional Integral Derivative) algorithm, the temperature error was controlled within ±0.5 °C.

**Figure 4:**
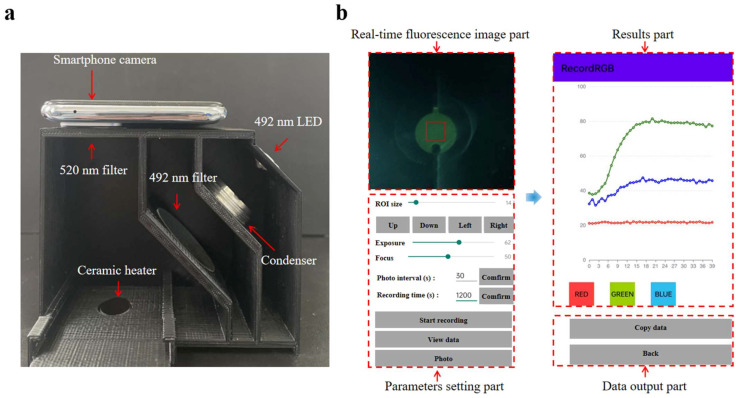
(**a**) The image of the real-time fluorescence detection system. (**b**) Screen capture images of the operation interface and result page of the custom-built Android application.

As depicted in Figure 4a, the fluorescence detection system comprises five parts: (1) an LED light source (3 W, 492 nm) serving as the emission source, and (2) the optical path, which includes an excitation filter (40 mm × 40 mm, 492 nm), an emission filter (20 mm radius, 520 nm), and a condensing lens. An Android smartphone (K9s, Oppo, Shenzhen, China) equipped with a 64-megapixel camera was utilized to capture and analyze the fluorescence images using a custom-built Android application coded in Java (Figure 4b). The RGB values are derived from the region of interest (ROI) in the captured fluorescence images, with the average values calculated to ascertain the fluorescence intensities [39]. The detailed functionality of the application is presented in Figure S3. The detection box was designed using SolidWorks (Concord, MA, USA) and created with a 3D printer (Snapmaker Original, Snapmaker Co., Ltd., Shenzhen, China).

## 3. Results and Discussion

### 3.1. RPA Optimization

**Figure 5:**
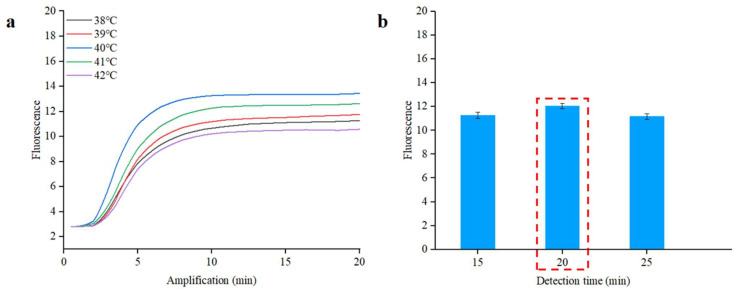
Real-time fluorescence optimal analysis of RPA for *S. typhimurium*. (**a**) Optimization of the reaction temperature. (**b**) Optimization of the reaction time. The inset represents real-time fluorescence curves of different reaction times.

To establish optimal conditions for the RPA reaction, we conducted a series of tests using *S. typhimurium* DNA at a concentration of 1.0 × 10^5^ copies/µL in the qPCR instrument. The reaction temperature significantly influences the reaction efficiency. To find the optimal temperature, we tested a range of temperatures from 38 °C to 42 °C for the RPA reaction. As illustrated in Figure 5a, the *C_p_* value at 40 °C is 2.5, which is lower than the values noted at other temperatures. Choosing an appropriate reaction time enhances the detection process’s efficiency. In order to optimize the RPA detection time, different reaction times (from 15 to 25 min) were applied to the RPA reaction. As demonstrated in Figure 5b, no notable differences were noted in *C_p_* values corresponding to different reaction times, but the highest fluorescence intensity was achieved at 20 min [40]. The real-time fluorescence curves of different reaction times are shown in Figure S7. Simultaneously, a complete amplification curve was observed within the initial 20 min. The results suggested that optimal amplification can be achieved at 40 °C for 20 min.

### 3.2. Sensitivity and Specificity of RPA

**Figure 6:**
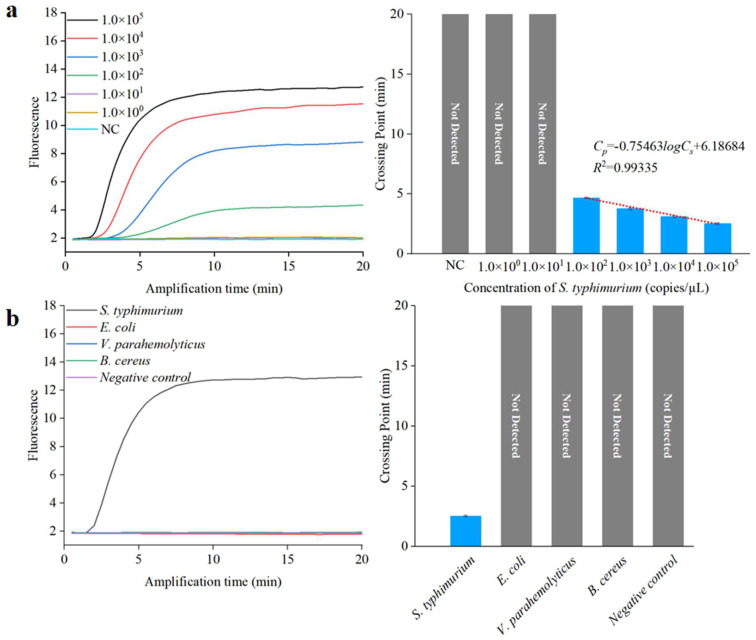
Sensitivity and specificity analysis using a qPCR instrument. (**a**) Sensitivity assessment of RPA. (**b**) Specificity assessment of RPA.

To achieve sensitive and specific detection of *Salmonella*, we assessed the analytical sensitivity and specificity of the RPA assay for *S. typhimurium*. We used 10-fold serial dilutions of *S. typhimurium* DNA to evaluate the detection limit. A standard curve for the RPA reactions was derived by plotting the *C_p_* value against the log 10 DNA copy number, as depicted in Figure 6a. The correlation coefficient and detection limit of the RPA method were 0.99 and 1.0 × 10^2^ copies/µL, respectively, indicating the RPA method could be employed for quantitative analysis. To demonstrate the specificity of the RPA method, three different non-target bacteria at a concentration of 1.0 × 10^5^ copies/µL, including *E. coli*, *V. parahemolyticus*, and *B. cereus*, were tested using this method. The results, shown in Figure 6b, indicate that only the target *S. typhimurium* yielded a fluorescent signal after amplification, signifying excellent sensitivity and specificity.

### 3.3. Mixing Efficiency

**Figure 7:**
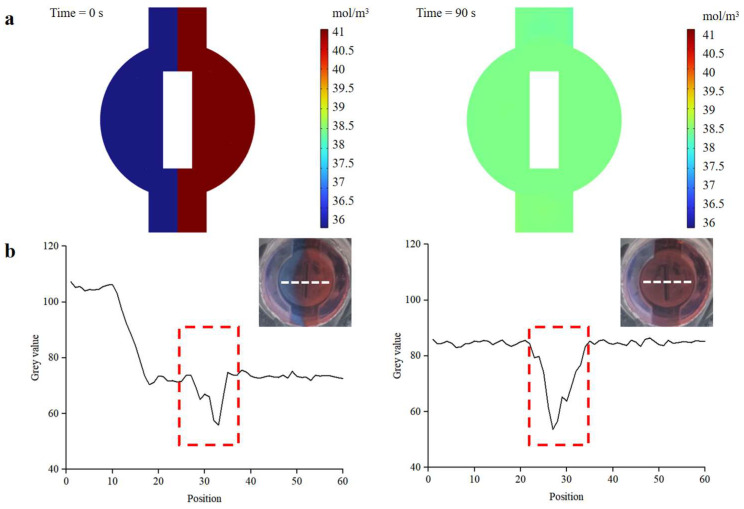
Mixing efficiency of the magnetic actuation mixer. (**a**) COMSOL simulation result of the mixer. (**b**) Images of the mixture before and after mixing. Mixing efficiency is analyzed using the gray value analysis method. The white dotted line in the insets is represented as the pixel point selected for gray value analysis. The red dotted line indicates the magnetic bar.

As demonstrated in Figure S4, the versatile valve was tested to ensure excellent fluid control, and its ability to open or close the fluid channel on-demand. Additionally, rapid and efficient mixing greatly influences detection efficiency and sensitivity. The versatile valve is designed as a combination of a valve and a magnetic actuation mixer, facilitating on-demand mixing and control of fluid flow. We conducted finite-element analysis simulations and mixing experiments to evaluate the performance of the active magnetic actuation mixer. The stirring speed of the magnetic bar was set at 360 °/s. As depicted in Figure 7a, the finite-element analysis simulations suggested that the magnetic bar accelerated the mixing process of the two dye solutions. To verify the mixer’s actual mixing effect, blue and red dyes were simultaneously injected into the mixer at the same flow rate (0.15 mL/s), and images during the mixing process were captured using a camera (EOS 5D Mark IV, Canon, Japan). The gray values of the images were analyzed using ImageJ software. As shown in Figure 7b, a clear interface appeared between the two dye solutions before mixing, and the gray values of the blue and red dye were 107.2 and 72.6, respectively (the red dotted line in the figure indicates the gray values of the magnetic bar). The gray values of the blue and red dyes converged to approximately 85, demonstrating that the active mixer enhanced the mixing efficiency.

### 3.4. Thermostatic Control

**Figure 8:**
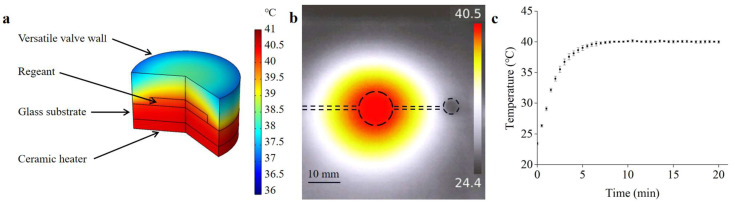
(**a**) The COMSOL simulation result of the temperature-constant state. (**b**) Thermal infrared image of the microfluidic chip. (**c**) The temperature variation of the reagents in the detection chamber.

Temperature and its distribution have a significant influence on RPA reaction efficiency. To validate the heating effects of the system, a finite-element analysis was performed to simulate the temperature and distribution of the reagents in the detection chamber. As can be seen in Figure 8a, the temperature in the detection chamber was evenly distributed and kept at 40 °C, which met the optimal reaction temperature for RPA detection. A temperature-measurement experiment was conducted to verify the simulation results. An infrared thermometer (UTi260, UNI-T, Dongguan, China) was used to measure the temperature distribution in the detection chamber. As shown in Figure 8b, the infrared image indicated that a uniform temperature distribution can be obtained, and the temperature was measured to be 40 °C. In addition, the temperatures of the reagents in the detection chamber were measured using a Pt100 thermal sensor. As shown in Figure 8c, the temperature of the detection chamber increased rapidly with heating time and became stable owing to the PID algorithm, indicating that the heater was suitable for providing an appropriate temperature for RPA detection.

### 3.5. Performance of the Microfluidic Biosensing System

**Figure 9:**
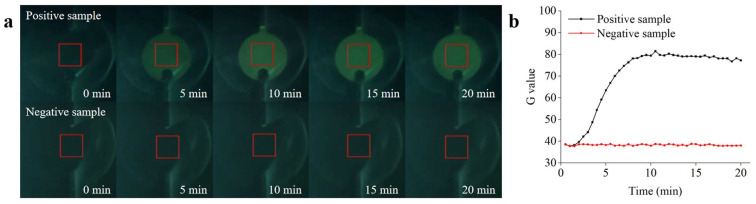
Real-time RPA analysis based on the microfluidic biosensing system. (**a**) Real-time fluorescence image of the amplification process. (**b**) Real-time fluorescence curve of the positive and negative samples recorded by the fluorescence detection system.

Experiments to determine the *Salmonella* detection performance of the microfluidic biosensing system were conducted under optimized conditions. The ceramic heater was preheated to achieve the optimal reaction temperature for the RPA. The reaction reagents were automatically transferred to the detection chamber immediately after sufficient mixing to initiate real-time RPA detection. To verify the performance of the microfluidic biosensing system, the positive sample (1.0 × 10^5^ copies/µL) and the negative control sample (containing all the reaction materials except target DNA) were detected. The acquisition setting parameters in the app were as follows: the acquisition interval and number of acquisitions of fluorescence images were set to 30 s and 40 s, respectively, and the rectangular ROI size was set to 12. As shown in Figure 9a, the fluorescence intensity first increased and then remained constant as the reaction progressed, which could be visualized by the naked eye (from the images acquired by the app) and quantified by the fluorescence detection system. No significant changes in the fluorescence intensity were observed in the negative sample. As shown in Figure 9b, an amplification curve for the positive sample was observed within 20 min, whereas no obvious fluorescence signals were observed for the negative control. These results are in general agreement with the real-time RPA analysis of the qPCR instrument, verifying the good detection ability of the microfluidic biosensing system.

**Figure 10:**
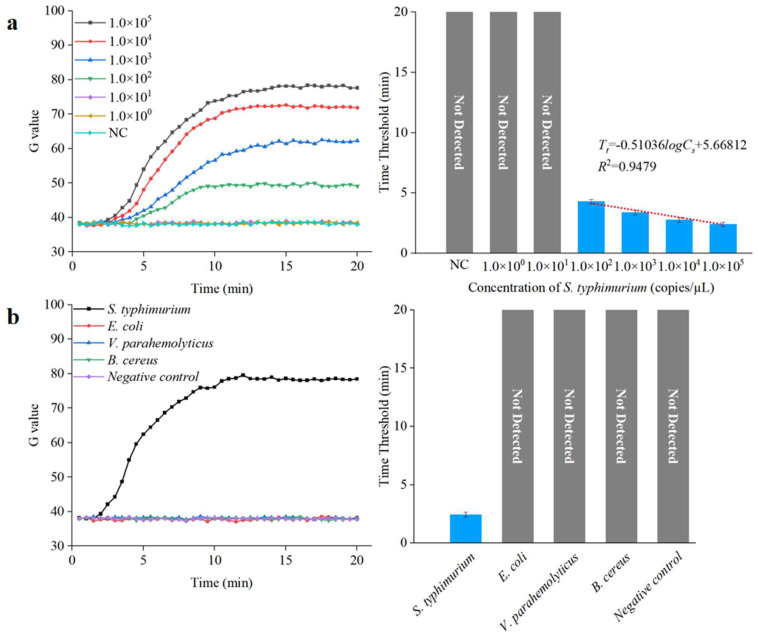
Sensitivity and specificity analysis of the microfluidic biosensing system. (**a**) Sensitivity of the microfluidic biosensing system (*N* = 3). (**b**) Specificity of the microfluidic biosensing system (*N* = 3).

To further verify the performance of this integrated microfluidic biosensing system for detecting unknown concentration of *Salmonella* in samples, three parallel tests on *S. typhimurium* DNA with different concentrations (1.0 × 10^0^ to 1.0 × 10^5^ copies/µL) were detected under optimal conditions. The threshold was calculated using the following equation:

(2) $$\mathrm{Threshold}=\bar{F_{n}}+3\sigma$$

where $\bar{F_{n}}$ is the average G value of negative control, and σ is the G value standard deviation of negative control. As shown in Figure 10a, the time threshold ($T_{t}$) decreased from 4.35 to 2.45 as the target DNA concentration ($C_{s}$) increased from 1.0 × 10^2^ to 1.0 × 10^5^ copies/µL. A good linear relationship can be found between the $T_{t}$ value and the logarithm of the $C_{s}$ value, with a correlation coefficient and detection limit of 0.94 and 1.0 × 10^2^ copies/µL, respectively; the expression is shown below:

(3) $$T_{t}=-0.51036\log C_{S}+5.66812$$

To demonstrate the specificity of this integrated microfluidic biosensing system, the target bacteria (*S. typhimurium*) and the non-target bacteria (*E. coli*, *V. parahemolyticus*, and *B. cereus*) at a concentration of 1.0 × 10^5^ copies/µL were detected under the optimal conditions. As shown in Figure 10b, the target *S. typhimurium* was observed after amplification, verifying that this integrated microfluidic biosensing system had good specificity.

## 4. Conclusions

In this study, we successfully developed an integrated microfluidic biosensing system using a microfluidic chip with a versatile valve, the RPA method for rapid and sensitive detection of *Salmonella*, and a customized real-time fluorescence detection system. A novel versatile valve was proposed as a combination of an active valve and a magnetic actuation mixer, which enabled on-demand mixing and fluid control to improve the detection efficiency of the microfluidic biosensing system. Further, a fluorescence detection system for the quantitative detection of RPA reactions was developed. Real-time fluorescence quantification results were acquired and analyzed using a custom-built smartphone application, which proved to be a perfect platform for on-site detection of pathogens. The proposed microfluidic detection biosensing system was able to detect *S. typhimurium* in 30 min with a detection limit of 1.0 × 10^2^ copies/µL. The integrated microfluidic biosensing system was evaluated with high sensitivity, good specificity, and feasible applicability for the detection of *Salmonella* and may provide great potential for the on-site detection of other foodborne pathogens.

## Data Availability

Data are contained within the article.

## Supplementary Materials

The following supporting information can be downloaded at: https://www.mdpi.com/article/10.3390/bios13080790/s1, Figure S1: (a) The photo of the integrated microfluidic biosensing system. (b) The photo of the real-time fluorescence detection system; Figure S2: Design scheme of Components A and B; Figure S3: Function introduction of the custom-built app. (a) Function introduction of the home page. (b) Function introduction of the test result page; Figure S4: Analysis of fluid control performance of the versatile valve. (a) Device setup for the versatile valve control performance test. (b) A voltage variation curve of different states of the versatile valve; Figure S5: Liquid residue analysis in the mixing chamber. The red dotted line indicates the liquid remaining around the mobile magnetic bar; Figure S6: The sensitivity test without the versatile valve; Figure S7: The real-time fluorescence curves of different reaction times; Video S1: Working principle of the versatile valve.
