# Supplementary material for: An Integrated Microfluidic Biosensing System Based on a Versatile Valve and Recombinase Polymerase Amplification for Rapid and Sensitive Detection of Salmonella typhimurium
Source: Biosensors (Basel). 2023 Aug 4;13(8):790. doi: 10.3390/bios13080790 (PMC10452558; doi:10.3390/bios13080790)
Supplement: Supplementary file 1 [file biosensors-13-00790-s001.zip › Revised Supplementary File.pdf]

Supplementary File

An Integrated Microfluidic Biosensing System Based on a Versatile Valve and Recombinase Polymerase Amplification for Rapid and Sensitive Detection of *Salmonella typhimurium*

Yan Jin[1], Jingyi Wang[1,2,*], Zhiqiang Wang[1], Peng Xiong[1], Jianing Cheng[3], Tongyu Xu[1,2,*]

[1]College of Information and Electrical Engineering, Shenyang Agricultural University, Shenyang 110866, China
[2]Liaoning Engineering Research Center for Information Technology in Agriculture, Shenyang 110866, China
[3]College of Bioscience and Biotechnology, Shenyang Agricultural University, Shenyang 110866, China

*Corresponding author: Jingyi Wang, Email: wangjingyi@syau.edu.cn; Tongyu Xu, Email: xutongyu@syau.edu.cn

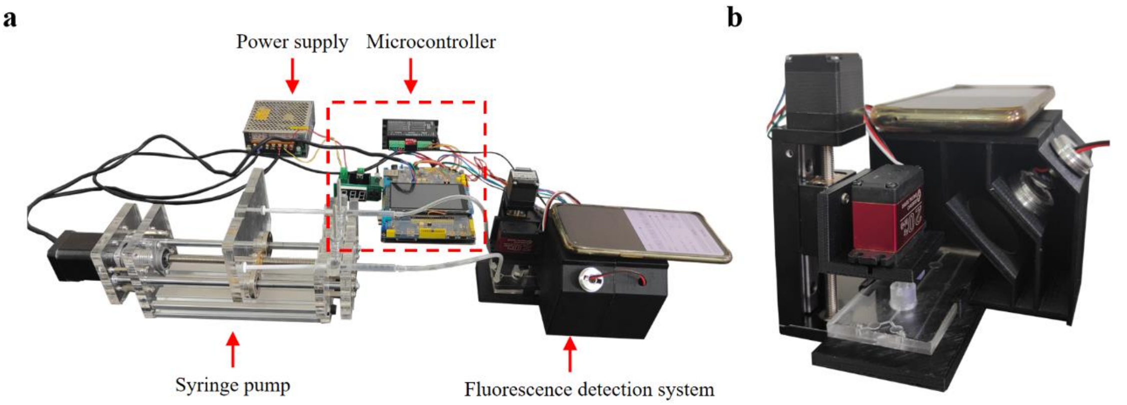

Figure S1. (a) The photo of the integrated microfluidic biosensing system. (b) The photo of the real-time fluorescence detection system.

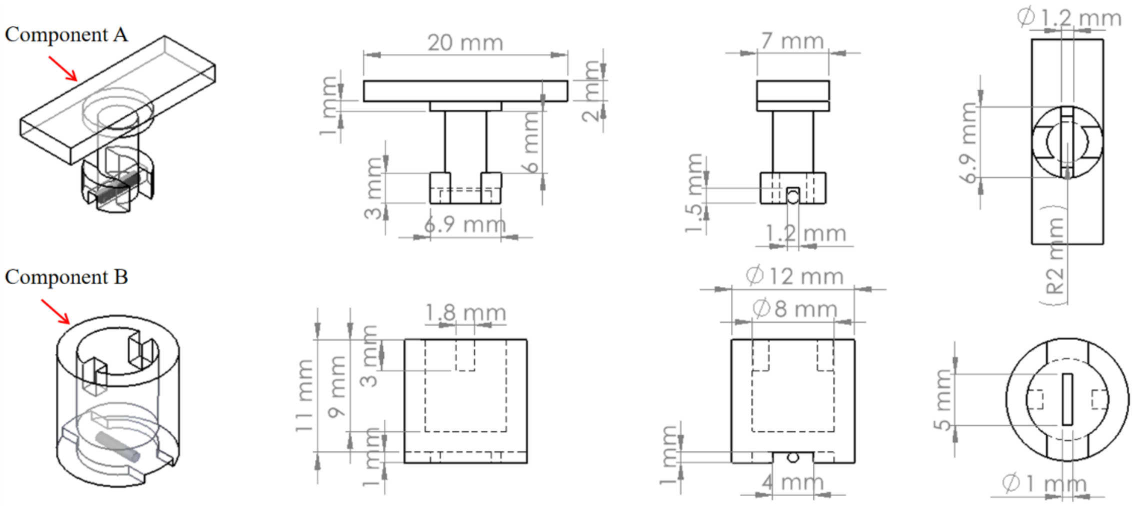

Figure S2. Design scheme of Components A and B.

Operation manual of the custom-built application

For a typical test, on the home page (operation interface) of the app, the user can set up the ROI parameters for fluorescence image analysis by clicking the "ROI size" and "ROI position" buttons. On the home page, the user can adjust the exposure and focus of the smartphone camera to capture sharp fluorescence images. The user can set the acquisition interval and number of acquisitions of fluorescence images by clicking the "Photo interval" and "Recording time" buttons. The user can click "Start recording" or "Photo" to obtain the fluorescence images, and the app can automatically calculate the average RGB value of the rectangular ROI. The test results can be viewed on the result interface by clicking the "View data" button. On the result interface, the user can click the "Copy data" button to export RGB data. The exposure and focus parameters selected for this work were set to 80 and 90, respectively.

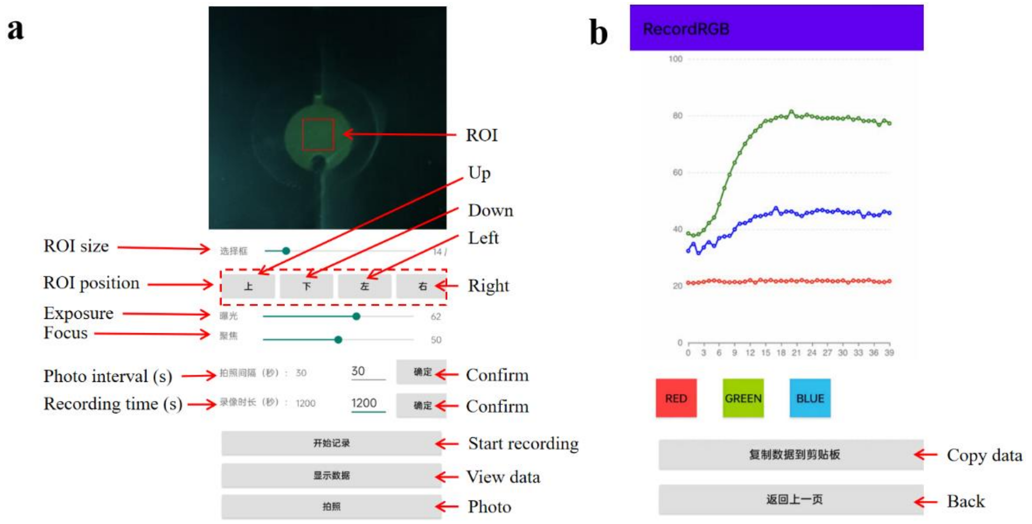

Figure S3. Function introduction of the custom-built app. (a) Function introduction of the home page. (b) Function introduction of the test result page.

Fluid control performance of the versatile valve

To evaluate the versatile valve's performance in controlling fluid flow, a 10% potassium carbonate solution was injected into the microfluidic chip and filled the entire fluid channel. A 10 V DC power supply was then connected to the inlet and outlet of the microfluidic chip and an oscilloscope (SDS1102A, SIGLENT, Shenzhen, China) was used to monitor the voltage changes in the fluid channel. As shown in Figure S4, the voltage was maintained at 10 V (Figure S4b) when the versatile valve was in the open position (ON state), nevertheless, a significant voltage descent occurred (from 10 V to 0 V) when the versatile valve was closed (OFF state), verifying the feasibility of the versatile valve.

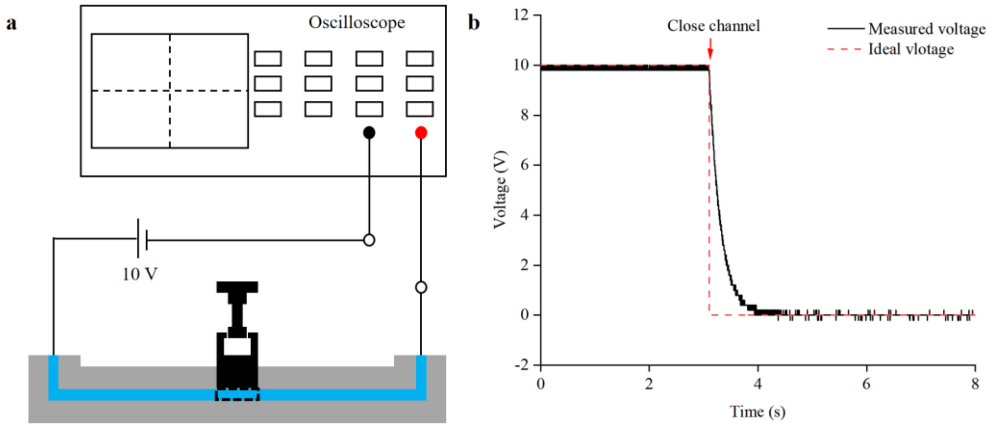

Figure S4. Analysis of fluid control performance of the versatile valve. (a) Device setup for the versatile valve control performance test. (b) A voltage variation curve of different states of the versatile valve.

Liquid residue test

The liquid residue test was conducted to test the impact of the mobile magnetic bar on the liquid in the mixing chamber. As shown in Figure S5, the liquid was thoroughly mixed in the mixing chamber and transferred to the detection chamber, with only a small amount of liquid remaining around the

mobile magnetic bar. According to the result of the RPA reaction, the remaining liquid or the mobile magnetic bar has no impact on the RPA reaction.

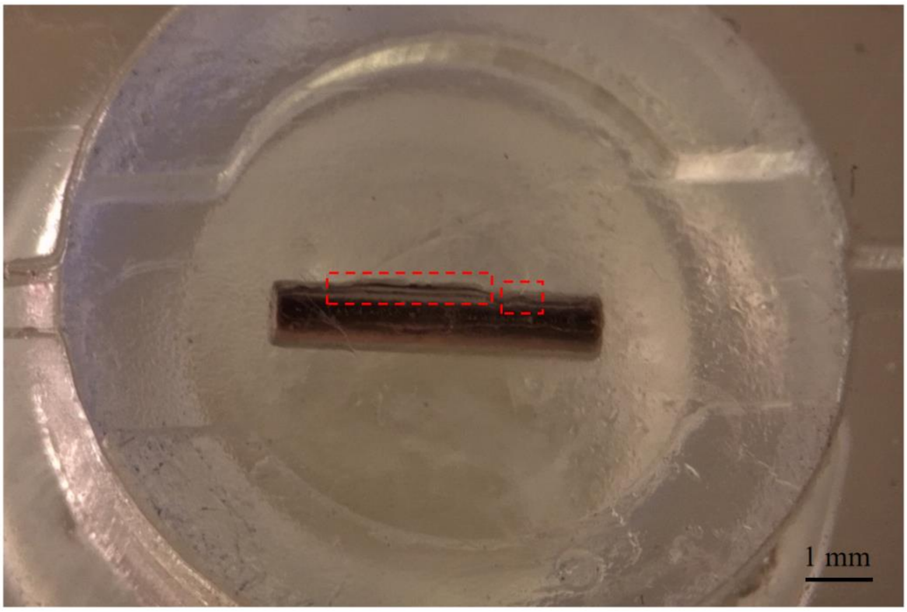

Figure S5. Liquid residue analysis in the mixing chamber. The red dotted line indicates the liquid remaining around the mobile magnetic bar.

Sensitivity test without the versatile valve

In order to verify the validity of the versatile valve, the sensitivity test without the versatile valve was conducted. Under the same conditions of the test with the microfluidic biosensing system, different concentrations (1.0×10^0 to 1.0×10^5 copies/μL) of the *S. typhimurium* DNA were detected. As shown in Figure S6, without sufficient mixing by the versatile valve, the amplification results of the 10^5 copies/μL and 10^4 copies/μL samples were unsatisfactory and the amplification results of other concentrations were not detected. The small dimensions of the microchannel and chambers will lead to the difficulty of natural (unassisted) mixing. Natural (unassisted) mixing in the microfluidic chips is based on molecular diffusion, which makes sufficient mixing a very slow process. Insufficient mixing will decrease the efficiency of the amplification reaction of RPA.

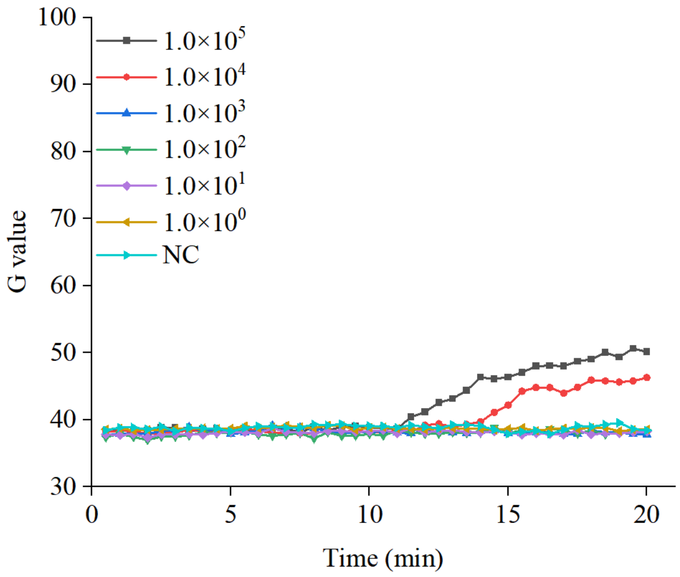

Figure S6. The sensitivity test without the versatile valve.

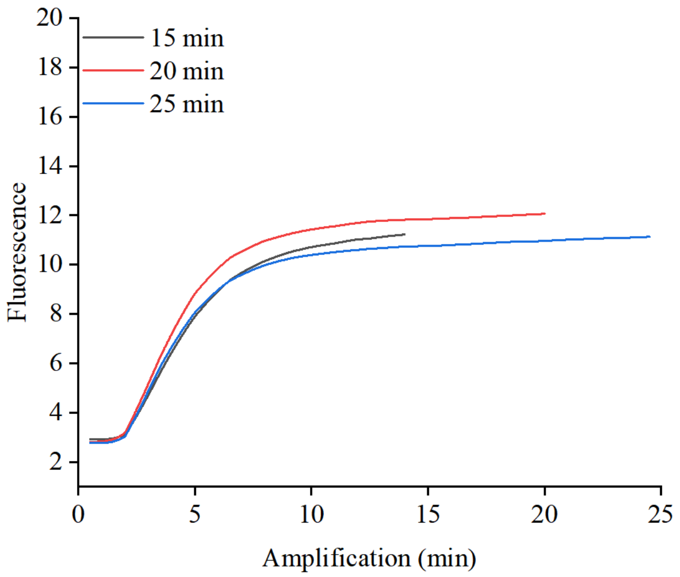

Figure S7. The real-time fluorescence curves of different reaction times.
