# Supplementary figures and images for: An Integrated Microfluidic Biosensing System Based on a Versatile Valve and Recombinase Polymerase Amplification for Rapid and Sensitive Detection of Salmonella typhimurium
Source: Biosensors (Basel). 2023 Aug 4;13(8):790. doi: 10.3390/bios13080790 (PMC10452558; doi:10.3390/bios13080790)

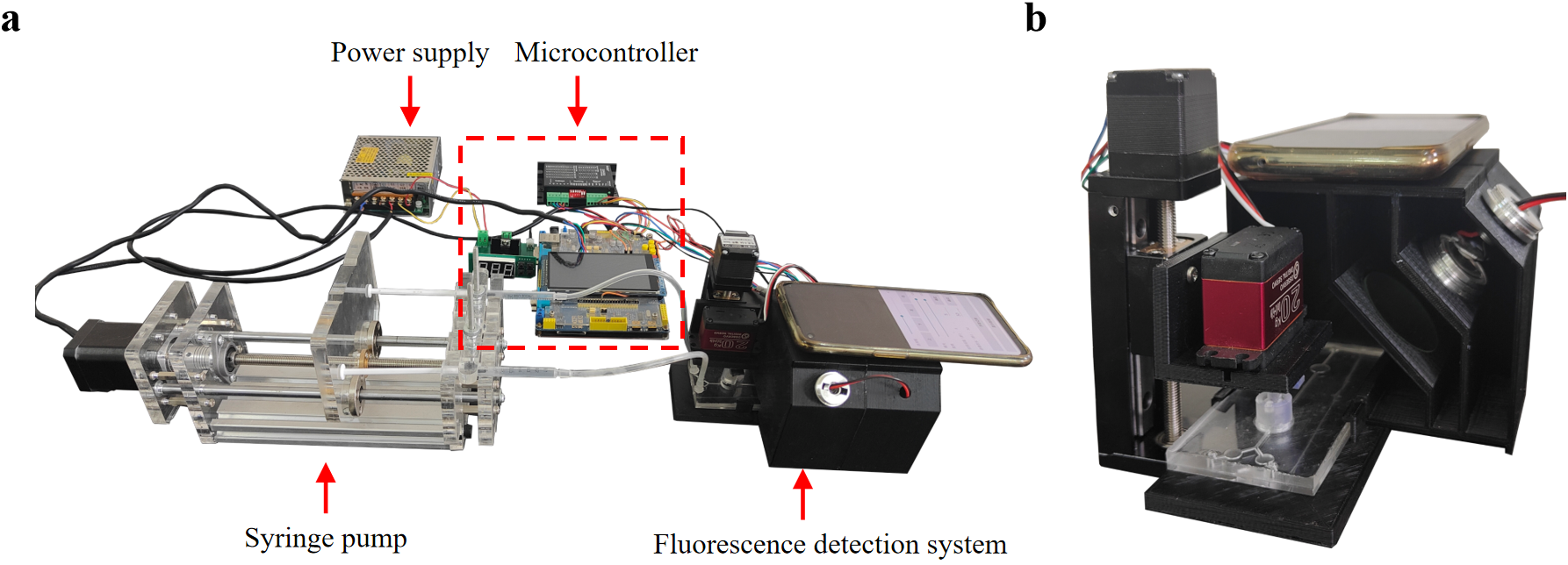

Supplement: Supplementary file 1 [file biosensors-13-00790-s001.zip › Figures/Figure S1.tif]

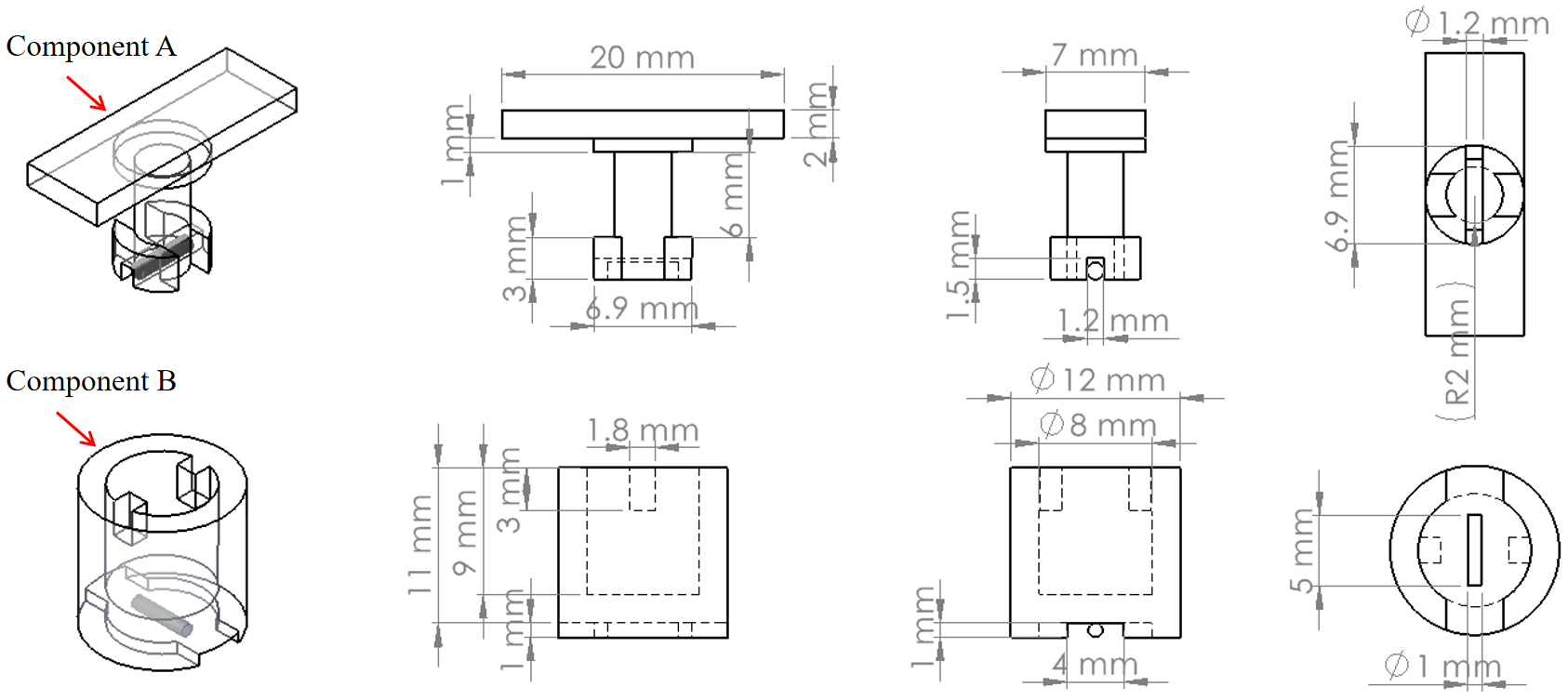

Supplement: Supplementary file 1 [file biosensors-13-00790-s001.zip › Figures/Figure S2.tif]

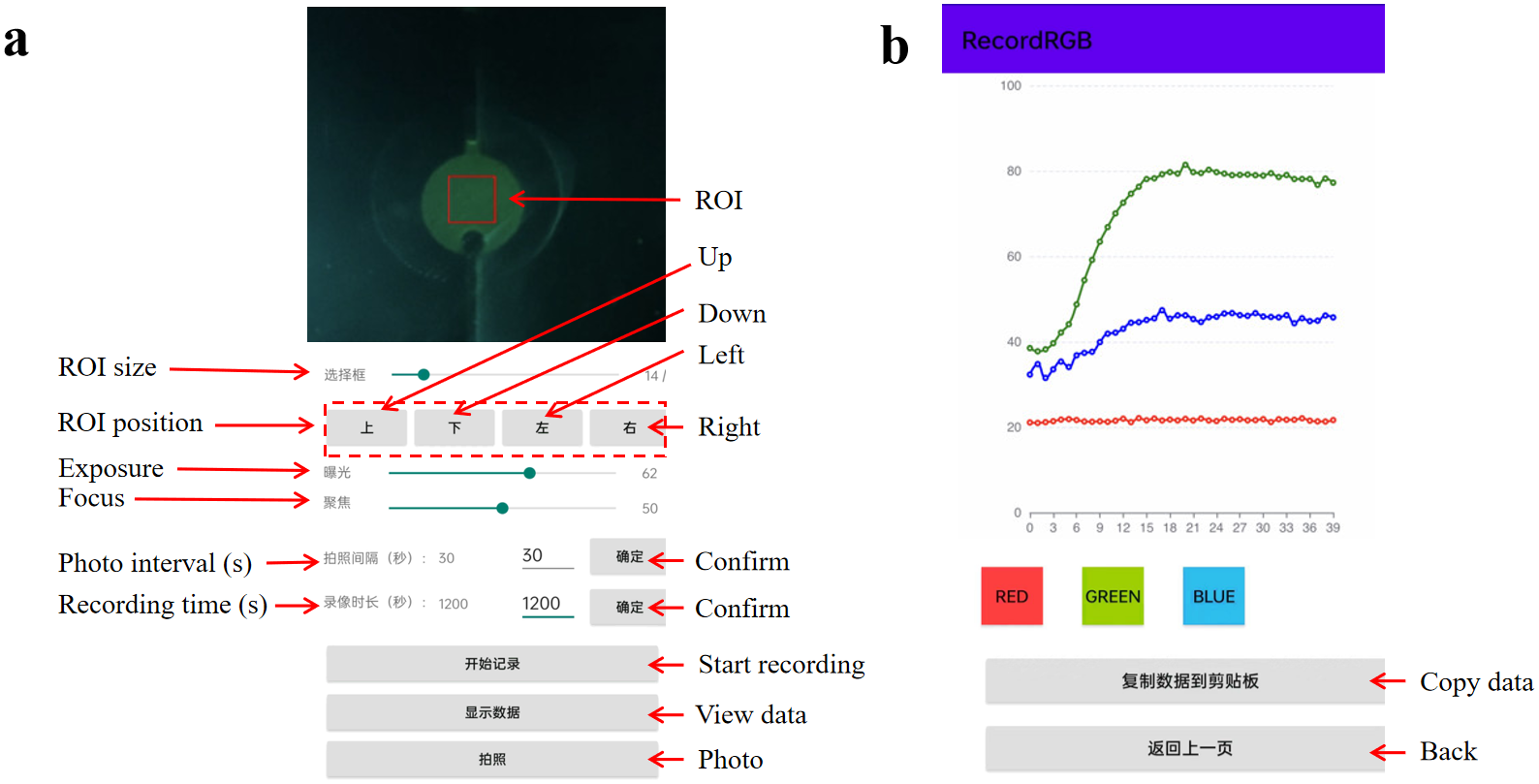

Supplement: Supplementary file 1 [file biosensors-13-00790-s001.zip › Figures/Figure S3.tif]

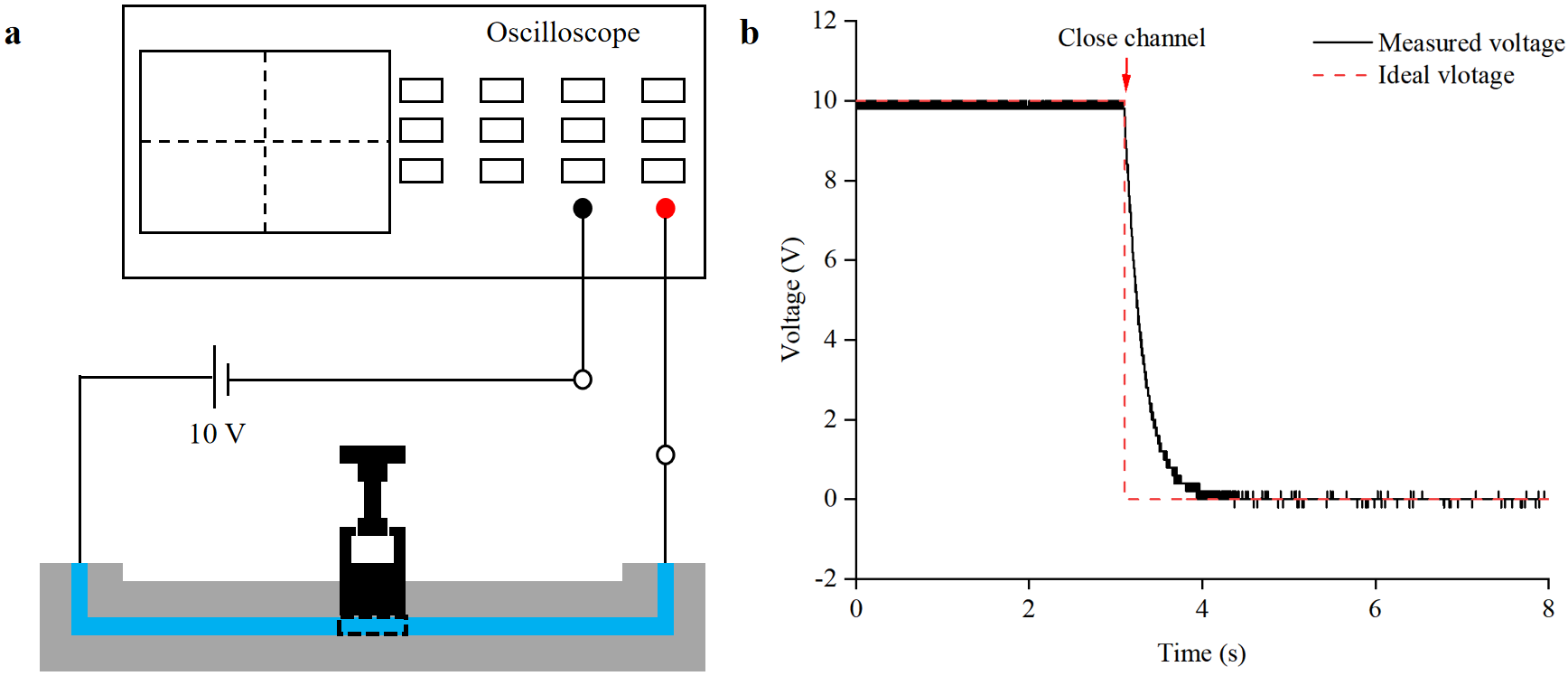

Supplement: Supplementary file 1 [file biosensors-13-00790-s001.zip › Figures/Figure S4.tif]

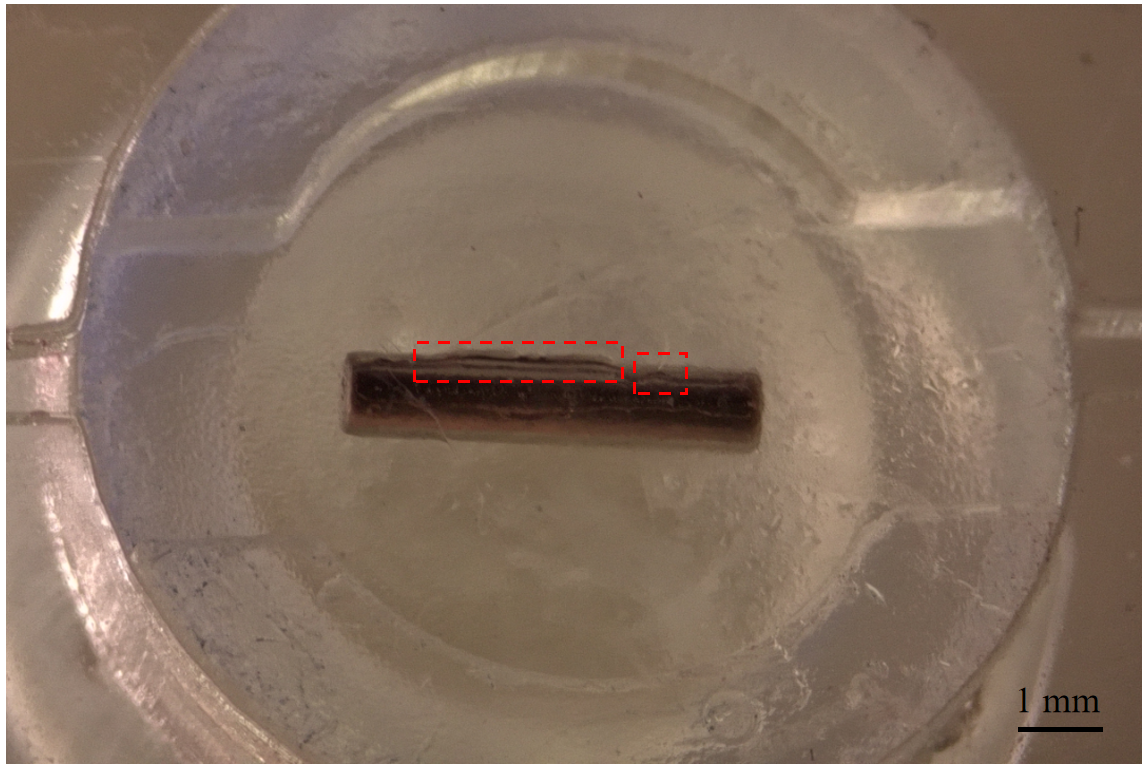

Supplement: Supplementary file 1 [file biosensors-13-00790-s001.zip › Figures/Figure S5.tif]

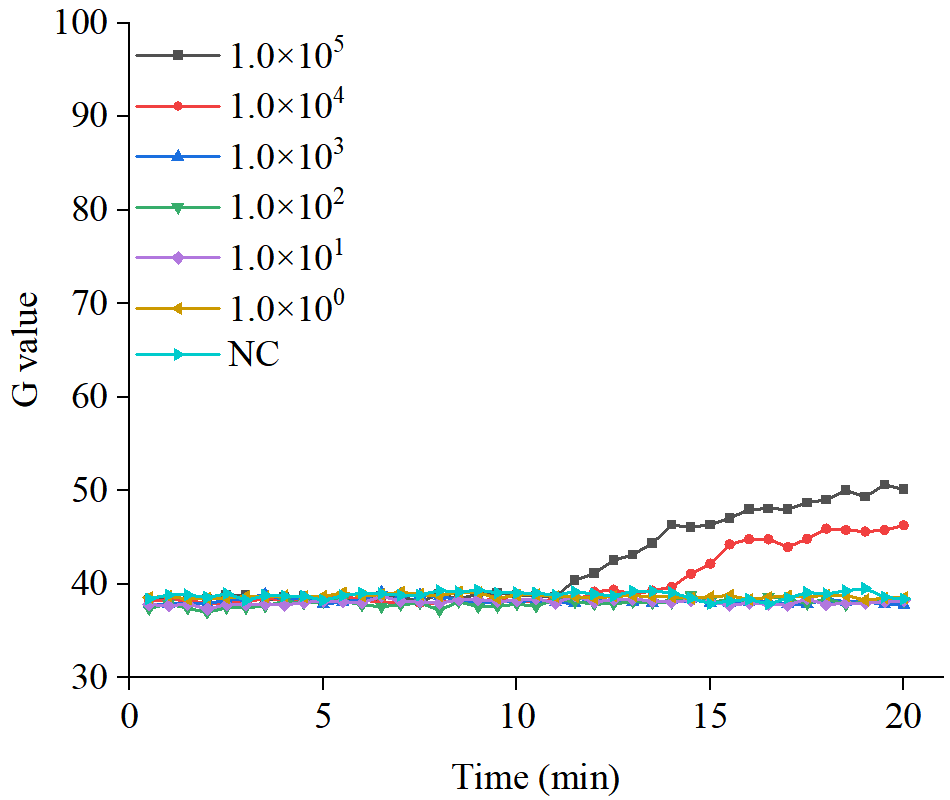

Supplement: Supplementary file 1 [file biosensors-13-00790-s001.zip › Figures/Figure S6.tif]

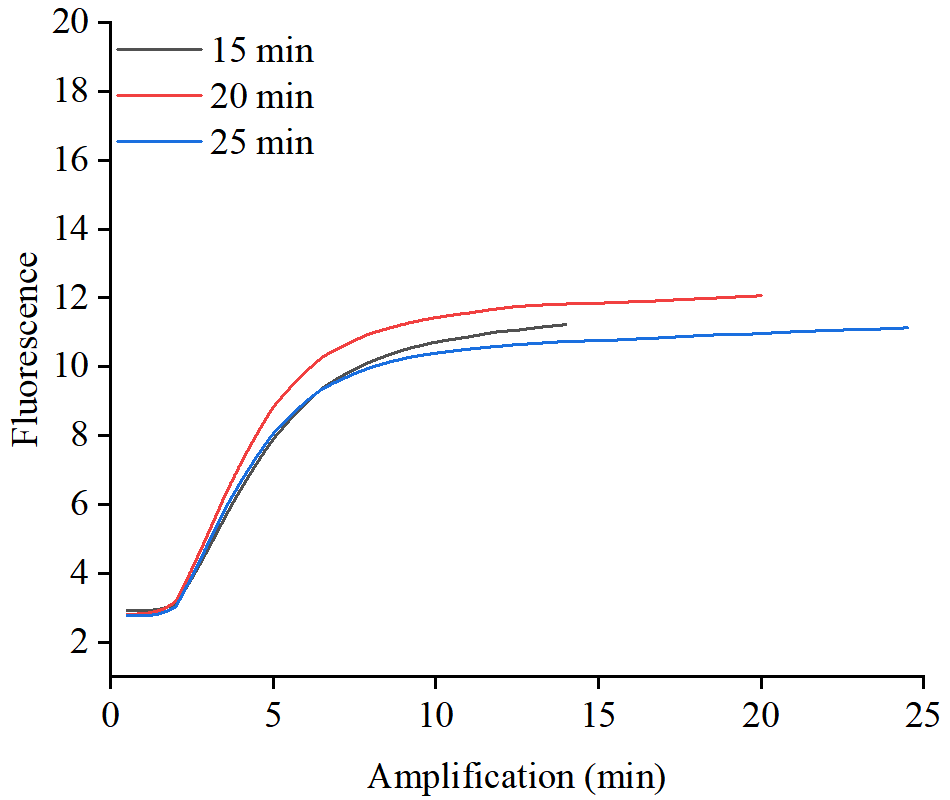

Supplement: Supplementary file 1 [file biosensors-13-00790-s001.zip › Figures/Figure S7.tif]
